# Prematurity and Prescription Asthma Medication from Childhood to Young Adulthood: A Danish National Cohort Study

**DOI:** 10.1371/journal.pone.0117253

**Published:** 2015-02-04

**Authors:** Anne Louise Damgaard, Bo Mølholm Hansen, René Mathiasen, Frederik Buchvald, Theis Lange, Gorm Greisen

**Affiliations:** 1 Department of Neonatology, Copenhagen University Hospital Rigshospitalet, Copenhagen, Denmark; 2 Department of Pediatric and Adolescent Medicine, Copenhagen University Hospital Rigshospitalet, Copenhagen, Denmark; 3 Dept. of Pediatric and Adolescent Medicine, Pulmonary Service, Copenhagen University Hospital Rigshospitalet, Copenhagen, Denmark; 4 Dept. of Biostatistics, University of Copenhagen, Copenhagen, Denmark; University Children’s Hospital Basel, SWITZERLAND

## Abstract

**Introduction:**

Preterm birth is associated with increased risk of asthma-like symptoms and purchase of prescription asthma medication in childhood. We investigated whether this association persists into adulthood and whether it is affected by accounting for neonatal respiratory morbidity (acute respiratory disease and bronchopulmonary dysplasia).

**Methods:**

A national cohort of all infants born in Denmark in the period 1980–2009 was included in this register study. Data on purchase of asthma medication (combination of inhaled β-2 agonists and other drugs for obstructive airway disease) in 2010–2011 were obtained from the Danish National Prescription Registry. Associations between gestational age (GA), neonatal respiratory morbidity and a cross-sectional evaluation of asthma medication purchase were explored by multivariate logistic regressions.

**Results:**

A full dataset was obtained on 1,790,241 individuals, 84.6% of all infants born in the period. Odds-ratios (95% CI) for the association between GA and purchase of asthma medication during infancy were: 3.86 (2.46–6.04) in GA 23–27 weeks, 2.37 (1.84–3.04) in GA 28–31 weeks and 1.59 (1.43–1.77) in GA 32–36 weeks compared to term infants with GA 37–42 weeks. Associations weakened in older age groups and became insignificant in young adults born extremely and very preterm with odds-ratios: 1.41 (0.63–3.19) and 1.15 (0.83–1.60) in GA 23–27 and 28–31 respectively. When adjusting for neonatal respiratory morbidity, the associations weakened but persisted both in childhood and adolescence.

**Conclusion:**

There was a strong dose-response association between gestational age and the purchase of prescription asthma medication in infancy and childhood. This association weakened during adolescence and was mostly non-significant in young adulthood. The increased risk of prescription asthma medication purchase in ex-preterm children could only partly be explained by neonatal respiratory morbidity.

## Background

Prematurity (gestational age (GA) <37 weeks) is associated with a high prevalence of neonatal respiratory distress compared to term birth. Nevertheless, most of these infants only require a few days or weeks of respiratory support and do not necessarily have any significant respiratory symptoms when they reach an age equivalent to term. However, the extremely preterm infants with GA 23–27 weeks are significantly more vulnerable. Their oxygen dependency tends to persist for many weeks and most of them develop some degree of bronchopulmonary dysplasia (BPD) [1]. BPD is followed by persisting lung abnormalities during the first years of life [2] alongside respiratory symptoms and bronchial hyper-responsiveness in both child- and adulthood [3–5].

Even though the vast majority of all preterm infants do not develop BPD, it has been shown that preterm birth is a risk factor for non-specific asthma-like symptoms in childhood [6–8]. However, it is not clear whether this increased risk of respiratory symptoms in childhood is linked to neonatal respiratory morbidity other than BPD or whether other predisposing factors associated with prematurity are involved. A Swedish register based study from 2011 found that the whole spectrum of prematurity is a risk factor for purchase of asthma controller medication during childhood and adolescence [8]. Since both bronchodilators and asthma controller medications are often prescribed to non-specific respiratory symptoms in infants and children, it is uncertain whether these symptoms should be expected to persist in adulthood. Another recent Swedish register based study found a significant increase in the purchase of prescription asthma medication in young adults born extremely preterm (GA under 28 weeks) but no increase in those born at GA 28–36 weeks compared to adults born at term, which suggests an outgrowing of the problem for the moderately preterm [9]. To our knowledge, no other studies have explored the effects of the entire spectrum of prematurity on respiratory symptoms and purchase of asthma medication in adulthood.

We therefore aimed to reassess the association between the full range of prematurity and the purchase of prescription asthma medication as a marker for the prevalence of a persistent disease burden of asthma-like symptoms across the age span from infancy to young adulthood. We included an assessment of the influence of neonatal respiratory morbidity on this association, in order to evaluate the potential link between neonatal respiratory morbidity and later respiratory symptoms. For this purpose, we conducted a register based study linking register data on an individual level in a Danish national cohort.

## Material and Methods

### Cohort

We conducted an observational retrospective register-based study of a national cohort of all infants born in Denmark in 1980–2009 with a cross-sectional follow-up in the period January 1^st^ 2010 to December 31^st^ 2011, when subjects were 0–31 years of age. The population in Denmark consists of 5.6 million inhabitants. A unique Central Personal Registration (CPR) number is assigned to all individuals at birth, to which all register entries on social and health services are consistently and unambiguously linked through the Civil Registration System [10,11].

Data were retrieved from Statistics Denmark in April 2012. The study population was divided into six separate age groups defined by age in years at follow-up (birth years): infants 0–2 (2009), preschool children 3–5 (2006–2008), children 6–11 (2000–2005), adolescents 12–17 (1994–1999), adults 18–24 (1987–1993) and 25–31 (1980–1986).

### Outcome

Data on all purchases of prescription medication from all non-hospital pharmacies during the two-year follow-up period 2010–2011 were obtained from the Danish National Prescription Registry. The register was established in 1994 and data are available through Statistics Denmark from 1995 [12]. Hospital pharmacy data are not retrievable on an individual basis from the Danish National Prescription Registry. To obtain a reliable marker of a significant burden of airway disease with asthma-like symptoms, including asthma, the outcome (“asthma medication”) was defined according to corresponding Anatomical Therapeutic Chemical (ATC) Classification System codes:

- either a combination of at least one purchase of inhaled selective β-2 receptor agonist (R03AC) AND at least two purchases of one of the following other drugs for obstructive airway disease: inhaled glucocorticoids (R03BA), inhaled anticholinergics (R03BB), theophyllines (R03DA), oral leukotriene-receptor antagonists (R03DC), systemic steroid (H02AB), a combination inhaler (R03AK)—or at least two purchases of a combination inhaler containing a long-acting β-2 receptor agonist and either glucocorticoids or anticholinergics (R03AK).

### Exposure variables

Our two exposures of interest were GA and neonatal respiratory morbidity.

### Gestational Age

GA was retrieved from the Medical Birth Registry. The variable was evaluated in full weeks and is reported to the register as assessed by a midwife and/or doctor. The register was established in 1973. In 1978 mid-wives’ reporting to the register was made mandatory by law. Before 1997 the reporting was made directly to the Danish Health and Medicines Authority. From 1997 onwards, the register has been based on a combination of a subset of the National Patient Register [13], the Civil Registration System [10] and the Causes of Death Register [14].

### Neonatal respiratory morbidity

Neonatal respiratory diagnoses were obtained from the National Patient Register during the half-year following the date of birth. The register was established in 1977 and contains information about patients from all public Danish hospitals. Data from an emerging private sector were included in the register from 2000 onwards. There are no private maternity or neonatal units in Denmark. Additionally, the register became the basis for payment of both public and private hospitals through the Diagnostic Related Group (DRG) system in 2000 [13].

The International Classification of Diseases ICD-10 system replaced the ICD-8 system in the register in 1994. Neonatal respiratory diagnoses were split into two groups. One group defined as “acute neonatal respiratory distress” included the diagnoses (ICD codes) of respiratory distress and transitory tachypnea of the neonate (ICD-8 776.290 / ICD-10 P22.1 and P22.8), respiratory distress syndrome (ICD-8 776.190 / ICD-10 P22.0) and neonatal emphysema (ICD-8 776.200 / ICD-10 P25.0). The second group was defined as “BPD” or chronic neonatal respiratory disease. It included the diagnoses of pulmonary fibrosis (ICD-8 517.010) and bronchopulmonary dysplasia (ICD-10 P27.1). The ICD-8 diagnosis of pulmonary fibrosis given in the neonatal period is assumed to be equivalent to the ICD-10 diagnosis BPD. In the following, the term BPD will be referring to both the ICD-8 and ICD-10 diagnoses.

### Covariates

Well-known risk factors for persistent respiratory symptoms, which were available in the registers, were also included in the multivariate analyses as potential confounding or mediating variables as follows.

### Birth-related

The Medical Birth Registry provided data on the following birth-related exposure variables in addition to GA:

- birth weight (BW), from 1979 in 10-grams groups and from 1991 in four digits. Considering the collinearity between GA and BW, the variable small-for-gestational age (SGA) was used in our analyses instead of BW. SGA was defined as a BW lower than -2SD from the reference value [15]
- sex
- older siblings
- multiple birth
- delivery method, i.e. cesarean section (subdivided into emergency and elective sections from 1982 onwards) or natural birth
- maternal smoking during pregnancy (data provided to the midwife at first antenatal care visit, available through Statistics Denmark from 1991 [16]).

### Socio-economic

Data on maternal educational level at the time of birth were obtained from the Register of Education of the Population. The data are provided through Statistics Denmark from 1981 in collaboration with the Danish Ministry of Education [17]. They are based on the Student Register from Statistics Denmark (updated annually) and include supplemental information from other authorities such as the Danish Agency for International Education and the Registered Healthcare Professionals Register. Educational level was evaluated according to the highest achieved education classified by the International Standard Classification of Education 2011 (ISCED) [18]. It was considered an indicator of socioeconomic status. ISCED levels were grouped into three groups:

- group 1 included ISCED levels 0–2: early childhood educational development, pre-primary education, primary education and lower secondary education.
- group 2 included ISCED levels 3–4: upper secondary education and post-secondary non-tertiary education.
- group 3 included ISCED levels 5–8: short-cycle tertiary education, Bachelor’s or equivalent level, Master’s or equivalent level, Doctor or equivalent level.

For children born in 1980, maternal education level was retrieved from 1981 data. When data on maternal education were missing, data on paternal education were used instead.

### Maternal asthma and atopic medication

Maternal asthma medication was defined as described for the cases. Maternal atopic medication was defined as at least one purchase of nasal steroid spray (R01AD), facial steroid cream group I (D07AA) or steroid cream group III (D07AC), all of which required a prescription. Since no medication data were available before 1995, maternal medication purchase was evaluated during the follow-up period in 2010–2011. Thus, it was not an indicator of maternal medication use during pregnancy but of asthmatic and atopic hereditary predisposition.

### Death before follow-up

Deaths were obtained from the Causes of Death Register, which contains detailed registration of all deaths in Denmark. The registration of Death Certificates was computerized in 1970 and since 2007 the certificates have been systematically issued and reported electronically. The register is matched with data from the Civil Registration System to ensure its validity [14].

### Statistics

Logistic regression analyses were performed in four setups with purchase of prescription asthma medication as the outcome variable:

- Unadjusted.
- Model 1: Adjusted for birth-related and socio-economic variables: sex, SGA, older siblings, multiple birth, maternal educational level, caesarean section, maternal asthma medication, maternal atopy medication and maternal smoking.
- Model 2: As model 1 and in addition including acute neonatal respiratory distress.
- Model 3: As model 2 and including BPD, i.e. all neonatal respiratory morbidities.

Only subjects with complete data were included in these analyses. SAS 9.3, SAS Institute was used for all statistical analyses.

### Ethics and data availability

Data extraction for the present study has been approved by the Danish Data Protection Agency and the Danish Health and Medicines Authority (Jr.no 2007–41–0806). Data were processed in accordance with Denmark’s Act on Processing of Personal Data, which allows linkages in this type of study without individual ethical approval.

The anonymized data are available from Statistics Denmark. Access can be granted to Danish research environments that are pre-approved by Statistics Denmark or to foreign researchers with an affiliation to a Danish authorized environment. Whilst we cannot guarantee such an access, we can assist interested institutions in any request in this context.

## Results

### Study population

**Fig 1:**
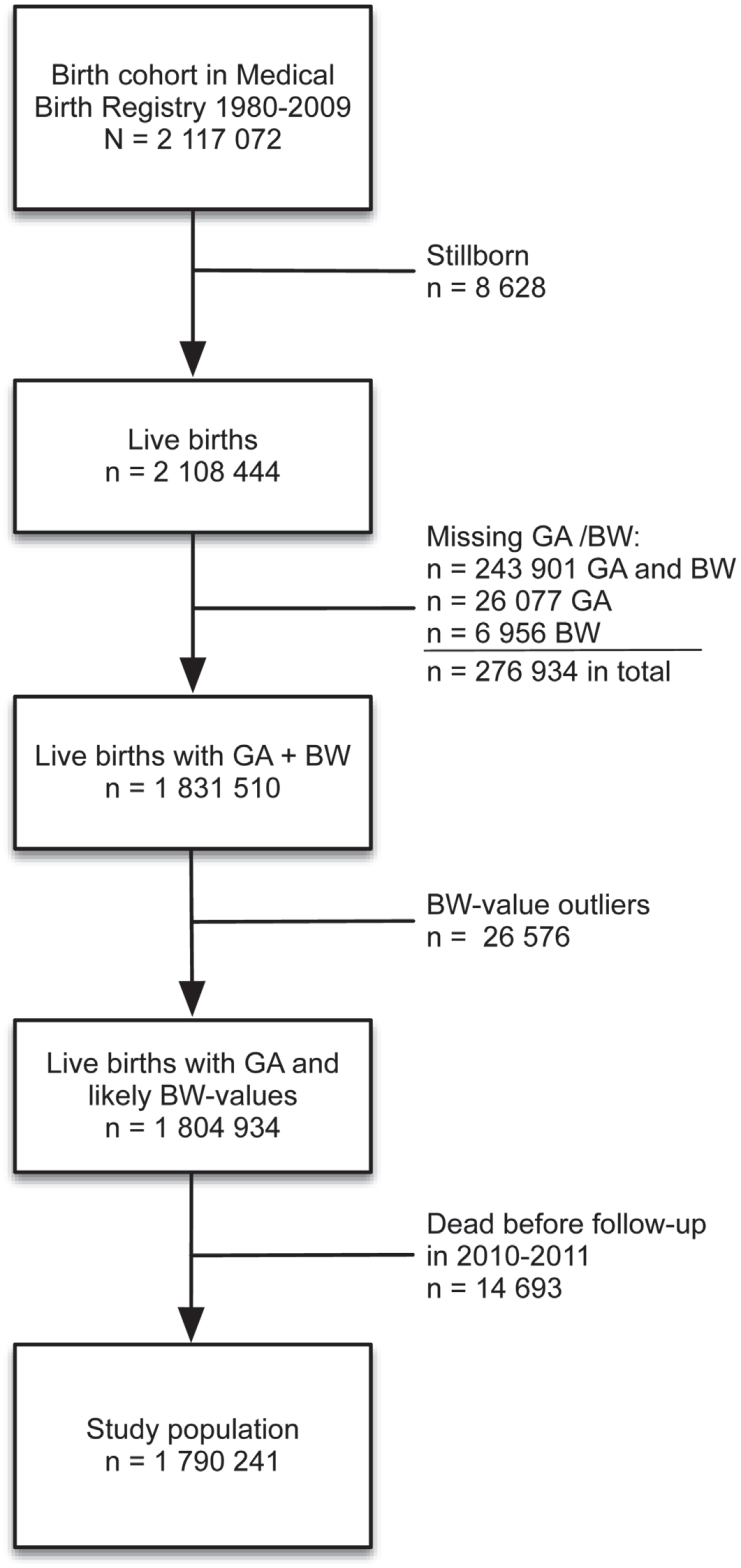
Exclusions from national birth cohort to study population. GA: gestational age, BW: birth weight.

Our study population of 1790241 individuals constituted 84.6% of the total of 2,117,072 individuals born in Denmark between January 1^st^ 1980 and December 31^st^ 2009. The missing 326,831 (15.4%) individuals were excluded due to stillbirth, missing data on either GA, BW or both GA and BW, BW-values differing +/- more than 3 standard deviations from reference values published by Marsál et al. [15] or due to death before follow-up (Fig. 1). The 3SD limit to BW-values was set in order to minimize the risk of including coding errors in the study population. Data on GA and BW were more likely to be missing in the early years of the cohort due to changes in the procedure of reporting weight and gestational age to the Medical Birth Registry in 1978 and 1979. The reported mean birth weight remained similar in individuals with and without data on GA during this period [19]. From 1982 onwards, GA and BW data were missing in fewer than 27% of individuals and this proportion declined for each year.

In the study population, 96,778 (5.4%) of the individuals were born preterm: 2,247 extremely preterm (GA 23–27), 9,626 very preterm (GA 28–31) and 84,905 moderately preterm (GA 32–36). The sex distribution was 917,010 males (51.2%) to 873,321 females (48.8%). The number of individuals included in each age group is indicated in Table 1.

**Table 1:** Prevalences of individuals having purchased prescription asthma medication according to gestational age and age groups in crude numbers and %.

	0–2 years (n = 61492)		3–5 years (n = 189215)		6–11 years (n = 378430)		12–17 years (n = 391192)		18–24 years (n = 424955)		25–31 years (n = 344957)	
GA in weeks	Prevalence	In %	Prevalence	In %	Prevalence	In %	Prevalence	In %	Prevalence	In %	Prevalence	In %
23–27 (n = 2247)	n = 26/98	26.5	n = 55/309	17.8	n = 51/681	7.5	n = 27/521	5.2	n = 15/434	3.5	n = 6/204	2.9
28–31 (n = 9626)	n = 76/419	18.1	n = 199/1207	16.5	n = 121/2347	5.2	n = 90/2124	4.2	n = 49/2034	2.4	n = 36/1495	2.4
32–36 (n = 84905)	n = 416/3205	13.0	n = 982/10334	9.5	n = 874/20936	4.2	n = 679/18472	3.7	n = 499/18248	2.7	n = 326/13710	2.4
37–45 (n = 1693463)	n = 4947/57770	8.6	n = 10363/177365	5.8	n = 10856/354466	3.1	n = 11317/370075	3.1	n = 8814/404239	2.2	n = 6919/329548	2.1

GA: gestational age.

### Descriptive analyses

A description of the proportions of each medication category purchased by individuals meeting our outcome definition criteria (purchase of at least one inhaled β-2 agonist *and* at least two other anti-asthmatic drugs *or* at least two purchases of combination inhalers) is available in S1 Table. Among these individuals, the medication proportions for short-acting β-2 agonists, inhaled glucocorticoids and oral leukotriene antagonists tended to be higher in those born preterm. On the contrary, long-acting β-2 agonists, combination inhalers and systemic glucocorticoids tended towards higher proportions in individuals born at term. We performed Chi-squared tests for each ATC-group to analyze these differences in proportions. The differences were highly significant for short-acting β-2 agonists, combination inhalers, inhaled glucocorticoids and oral leukotriene antagonists, all of which remained significant after Bonferroni correction.

The prevalence of individuals having purchased prescription asthma medication decreased with increasing age in both the term and preterm groups. At all ages, the prevalence was higher in the preterm groups compared to the term group and increasing with decreasing GA (Table 1).

**Fig 2:**
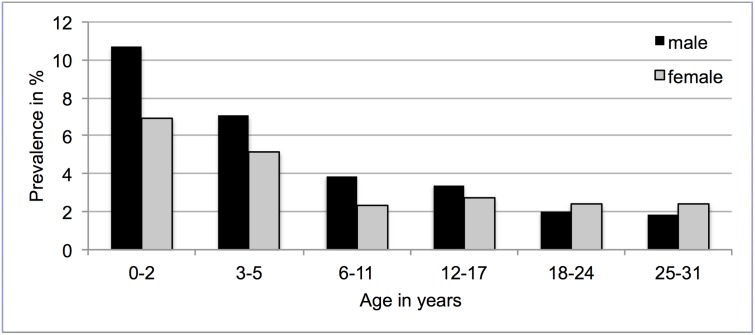
Purchase of asthma medication in age groups by gender. Prevalences in %.

The overall gender distribution of asthma medication purchase evolved across the age groups with high prevalences in boys during early childhood (10.7% (95% CI 10.4–11.1) compared to 7.0 (CI 6.7–7.3) in girls at 0–2 years of age), and higher prevalences in women in adulthood (2.4% (CI 2.3–2.5) compared to 1.8% (CI 1.8–1.9) in men at 25–31 years of age) (Fig. 2).

BPD was predominantly present in the extremely preterm and very preterm. The extremely preterm demonstrated high BPD prevalences, ranging from 34.7% (CI 30.7–38.8) to 43.6% (CI 39.9–47.3) in the young age groups. In the adult age groups, BPD prevalence in the extremely preterm was considerably lower: 4.8% (CI 2.9–6.9) in 18–24 years and 8.3% (CI 4.5–12.1) 25–31 years. Acute neonatal respiratory disease was present in all preterm groups, but was overrepresented in the extremely preterm with prevalences from 72.3% (CI 68.1–76.6) in 18–24 years to 89.0% (CI 85.5–92.5) in 3–5 years (Table 2). Acute neonatal respiratory disease also demonstrated a gradual increase in prevalence over time in individuals born at term (from 0.8% (CI 0.8–0.9) in 25–31 year-olds to 4.0% (CI 3.8–4.2) in 0–2 year-olds).

**Table 2:** Neonatal respiratory diagnoses according to GA for each age group.

	0–2 years (n = 61492)		3–5 years (n = 189215)		6–11 years (n = 378430)		12–17 years (n = 391192)		18–24 years (n = 424955)		25–31 years (n = 344957)	
GA in weeks	% with BPD	% with other^1^	% with BPD	% with other^1^	% with BPD	% with other^1^	% with BPD	% with other^1^	% with BPD	% with other^1^	% with BPD	% with other^1^
23–27 (n = 2247)	42.9	74.5	43.0	89.0	43.6	80.5	34.7	73.9	4.8	72.4	8.3	72.6
28–31 (n = 9626)	2.9	73.0	4.1	75.0	5.5	68.8	5.3	53.9	0.4	59.5	1.2	58.3
32–36 (n = 84905)	0.0	24.9	0.0	23.3	0.0	19.7	0.1	15.6	0.0	16.9	0.0	18.3
37–45 (n = 1693463)	0.0	4.0	0.0	3.2	0.0	2.3	0.0	1.5	0.0	1.1	0.0	0.8

GA: gestational age, BPD: bronchopulmonary dysplasia, CI: confidence interval.

^1^: acute neonatal respiratory disease including respiratory distress, respiratory distress syndrome, transitory tachypnea of the neonate and neonatal emphysema.

### Main analyses

Table 3 shows unadjusted odds ratios (OR) as well as ORs for models 1 and 3 for each variable, compared to the most frequently occurring value of the variable. Interaction with sex was tested in all models. A detailed description of missing values in all variables included in these analyses is available in S2 Table.

**Table 3:** Logistic regression analyses on associations with purchase of prescription asthma medication.

Total n = 1790241			0–2 years (n = 61492)			3–5 years (n = 189215)			6–11 years (n = 378430)			12–17 years (n = 391192)			18–24 years (n = 424955)			25–31 years (n = 344957)		
			Unadjusted OR (95% CI)	Model 1^1^ OR (95% CI) n = 58219	Model 3^2^ OR (95% CI)	Unadjusted OR (95% CI)	Model 1^1^ OR (95% CI) n = 179595	Model 3^2^ OR (95% CI)	Unadjusted OR (95% CI)	Model 1^1^ OR (95% CI) n = 365469	Model 3^2^ OR (95% CI)	Unadjusted OR (95% CI)	Model 1^1^ OR (95% CI) n = 367225	Model 3^2^ OR (95% CI)	Unadjusted OR (95% CI)	Model 1^1^ OR (95% CI) n = 418750	Model 3^2^ OR (95% CI)	Unadjusted OR (95% CI)	Model 1^1^ OR (95% CI) n = 338494	Model 3^2^ OR (95% CI)
GA in weeks	23–27^3^	(n = 2247)	3.86 (2.46–6.04)	3.27 (1.96–5.48)	2.58 (1.34–4.96)	3.49 (2.61–4.67)	3.34 (2.43–4.59)	1.83 (1.22–2.76)	2.56 (1.93–3.41)	2.30 (1.66–3.19)	1.54 (1.02–2.32)	1.73 (1.18–2.55)	1.57 (1.01–2.45)	1.19 (0.72–1.99)	1.61 (0.96–2.69)	1.56 (0.93–2.61)	1.44 (0.84–2.46)	1.41 (0.63–3.19)	1.39 (0.61–3.13)	0.83 (0.33–2.11)
	28–31^3^	(n = 9626)	2.37 (1.84–3.04)	2.23 (1.70–2.92)	2.00 (1.51–2.65)	3.18 (2.73–3.71)	2.99 (2.52–3.55)	2.38 (1.98–2.86)	1.72 (1.43–2.07)	1.62 (1.33–1.98)	1.30 (1.05–1.61)	1.40 (1.13–1.73)	1.34 (1.06–1.68)	1.17 (0.92–1.49)	1.11 (0.83–1.47)	1.02 (0.76–1.37)	0.96 (0.71–1.30)	1.15 (0.83–1.60)	1.10 (0.79–1.55)	0.99 (0.69–1.42)
	32–36^3^	(n = 84905)	1.59 (1.43–1.77)	1.52 (1.35–1.71)	1.48 (1.31–1.67)	1.69 (1.58–1.81)	1.67 (1.55–1.81)	1.59 (1.47–1.71)	1.38 (1.29–1.48)	1.37 (1.27–1.48)	1.30 (1.20–1.40)	1.21 (1.12–1.31)	1.18 (1.08–1.28)	1.14 (1.04–1.24)	1.26 (1.15–1.38)	1.23 (1.12–1.35)	1.21 (1.09–1.33)	1.14 (1.02–1.27)	1.13 (1.01–1.27)	1.12 (0.99–1.26)
Female sex		(n = 873231)	0.62 (0.59–0.66)	0.62 (0.59–0.66)	0.63 (0.59–0.66)	0.71 (0.68–0.74)	0.71 (0.68–0.74)	0.72 (0.69–0.74)	0.60 (0.58–0.62)	0.60 (0.58–0.62)	0.60 (0.58–0.63)	0.80 (0.77–0.83)	0.81 (0.78–0.84)	0.81 (0.78–0.84)	1.24 (1.19–1.29)	1.24 (1.19–1.29)	1.24 (1.19–1.29)	1.31 (1.25–1.38)	1.31 (1.25–1.37)	1.31 (1.25–1.38)
SGA		(n = 53641)	1.14 (0.94–1.37)	0.95 (0.78–1.15)	0.95 (0.78–1.15)	1.33 (1.18–1.50)	1.12 (0.99–1.27)	1.11 (0.98–1.26)	1.29 (1.15–1.44)	1.20 (1.07–1.35)	1.20 (1.07–1.34)	1.17 (1.05–1.29)	1.13 (1.02–1.26)	1.13 (1.02–1.26)	1.19 (1.07–1.32)	1.10 (0.99–1.23)	1.10 (0.99–1.23)	1.04 (0.92–1.16)	1.00 (0.89–1.12)	1.00 (0.89–1.12)
No older siblings		(n = 810310)	1.11 (1.05–1.18)	1.10 (1.04–1.16)	1.10 (1.03–1.16)	1.16 (1.11–1.20)	1.15 (1.10–1.19)	1.14 (1.10–1.19)	1.13 (1.09–1.17)	1.11 (1.07–1.16)	1.11 (1.07–1.15)	1.15 (1.10–1.19)	1.15 (1.10–1.19)	1.15 (1.10–1.19)	1.09 (1.05–1.13)	1.10 (1.06–1.15)	1.10 (1.06–1.15)	1.06 (1.02–1.11)	1.07 (1.02–1.12)	1.07 (1.02–1.12)
Multiple birth		(n = 56431)	1.15 (1.01–1.31)	0.90 (0.77–1.04)	0.89 (0.77–1.04)	1.12 (1.03–1.23)	0.78 (0.70–0.86)	0.77 (0.70–0.85)	1.03 (0.94–1.13)	0.82 (0.74–0.90)	0.82 (0.74–0.90)	0.97 (0.88–1.07)	0.83 (0.74–0.93)	0.83 (0.74–0.93)	1.10 (0.97–1.25)	0.96 (0.84–1.10)	0.96 (0.84–1.10)	0.92 (0.77–1.09)	0.88 (0.73–1.05)	0.88 (0.74–1.06)
Maternal educational level^4^	2	(n = 629889)	1.03 (0.96–1.11)	1.08 (1.01–1.16)	1.08 (1.01–1.16)	1.03 (0.99–1.08)	1.06 (1.01–1.11)	1.05 (1.00–1.11)	0.98 (0.94–1.03)	0.99 (0.95–1.04)	0.99 (0.95–1.04)	1.00 (0.96–1.04)	1.01 (0.97–1.06)	1.01 (0.97–1.06)	1.02 (0.98–1.07)	1.04 (0.99–1.09)	1.04 (0.99–1.09)	1.00 (0.95–1.06)	1.03 (0.98–1.09)	1.03 (0.97–1.09)
	3	(n = 439903)	0.85 (0.79–0.91)	0.92 (0.86–0.99)	0.92 (0.86–1.00)	0.86 (0.82–0.91)	0.91 (0.87–0.96)	0.91 (0.87–0.96)	1.04 (0.99–1.09)	1.06 (1.01–1.11)	1.06 (1.01–1.11)	1.16 (1.11–1.21)	1.18 (1.12–1.24)	1.18 (1.12–1.24)	1.19 (1.13–1.26)	1.22 (1.16–1.29)	1.22 (1.16–1.29)	1.05 (0.99–1.12)	1.09 (1.02–1.16)	1.09 (1.02–1.16)
Cesarean section	elective	(n = 98614)	1.22 (1.11–1.34)	1.22 (1.11–1.34)	1.21 (1.10–1.34)	1.27 (1.19–1.35)	1.28 (1.20–1.37)	1.26 (1.18–1.35)	1.21 (1.13–1.29)	1.22 (1.14–1.31)	1.20 (1.12–1.29)	1.19 (1.10–1.29)	1.18 (1.08–1.28)	1.17 (1.08–1.27)	1.20 (1.09–1.31)	1.16 (1.06–1.27)	1.15 (1.05–1.26)	1.10 (1.03–1.18)^5^	1.08 (1.00–1.16)^5^	1.08 (1.00–1.16) ^5^
	emergency	(n = 162580)	1.32 (1.22–1.43)	1.16 (1.07–1.26)	1.15 (1.05–1.25)	1.33 (1.26–1.40)	1.13 (1.07–1.20)	1.11 (1.05–1.18)	1.26 (1.19–1.32)	1.13 (1.07–1.19)	1.11 (1.05–1.18)	1.22 (1.15–1.30)	1.16 (1.08–1.23)	1.15 (1.08–1.23)	1.16 (1.07–1.24)	1.10 (1.02–1.19)	1.10 (1.02–1.19)			
Maternal asthma medication		(n = 69456)	2.38 (2.08–2.73)	2.15 (1.86–2.48)	2.14 (1.86–2.47)	3.23 (2.98–3.49)	2.92 (2.69–3.17)	2.92 (2.69–3.17)	3.59 (3.37–3.84)	3.23 (3.02–3.46)	3.23 (3.02–3.46)	3.03 (2.84–3.23)	2.83 (2.65–3.03)	2.83 (2.65–3.02)	2.55 (2.38–2.73)	2.40 (2.23–2.57)	2.40 (2.23–2.57)	2.30 (2.14–2.48)	2.22 (2.06–2.40)	2.22 (2.06–2.39)
Maternal atopy medication		(n = 319471)	1.41 (1.32–1.50)	1.35 (1.27–1.45)	1.35 (1.26–1.45)	1.50 (1.44–1.57)	1.40 (1.33–1.46)	1.40 (1.33–1.46)	1.53 (1.47–1.60)	1.39 (1.33–1.45)	1.39 (1.33–1.45)	1.48 (1.42–1.55)	1.35 (1.29–1.41)	1.35 (1.29–1.41)	1.45 (1.38–1.52)	1.34 (1.27–1.40)	1.34 (1.27–1.40)	1.31 (1.24–1.39)	1.22 (1.15–1.29)	1.22 (1.15–1.29)
Maternal smoking		(n = 261613)	1.43 (1.33–1.54)	1.40 (1.30–1.52)	1.41 (1.30–1.52)	1.28 (1.22–1.35)	1.23 (1.17–1.30)	1.23 (1.17–1.30)	1.01 (0.96–1.06)	1.01 (0.96–1.06)	1.01 (0.96–1.06)	1.00 (0.96–1.05)	1.00 (0.96–1.05)	1.01 (0.96–1.05)						
BPD		(n = 1071)	4.11 (2.30–7.34)		1.33 (0.60–2.94)	5.30 (3.83–7.34)		2.14 (1.36–3.36)	3.23 (2.35–4.43)		1.43 (0.90–2.26)	2.27 (1.46–3.53)		1.41 (0.77–2.58)	1.43 (0.20–10.47)		1.07 (0.14–8.12)	8.98 (3.74–21.52)		9.23 (3.41–25.03)
Acute neonatal respiratory disease		(n = 52580)	1.55 (1.40–1.72)		1.16 (1.03–1.31)	1.83 (1.71–1.96)		1.32 (1.22–1.43)	1.66 (1.54–1.79)		1.35 (1.23–1.47)	1.42 (1.29–1.57)		1.24 (1.10–1.39)	1.22 (1.07–1.38)		1.11 (0.97–1.28)	1.13 (0.96–1.34)		1.05 (0.87–1.26)

OR: odds ratio, GA: gestational age, SGA: small-for-gestational age, BPD: bronchopulmonary dysplasia.

*Values in italic font*: p<0.05, values in regular font: p≥0.05.

^1^: Adjusted for sex, SGA, sibship, multiple birth, maternal educational level, cesarean section, maternal asthma medication, maternal atopy medication and maternal smoking (only available for age groups <18 years).

^2^: Adjusted for model 1 variables^1^ and neonatal respiratory morbidity (BPD + acute neonatal respiratory disease).

^3^: Compared to GA 37–42 weeks.

^4^: Compared to educational level 1. When maternal data were missing, paternal educational levels were used (in 2.1% of the cases in the variable).

^5^: Segregation between emergency and elective cesarean section not available before 1982. Values represents cesarean section compared to natural delivery.

**Fig 3:**
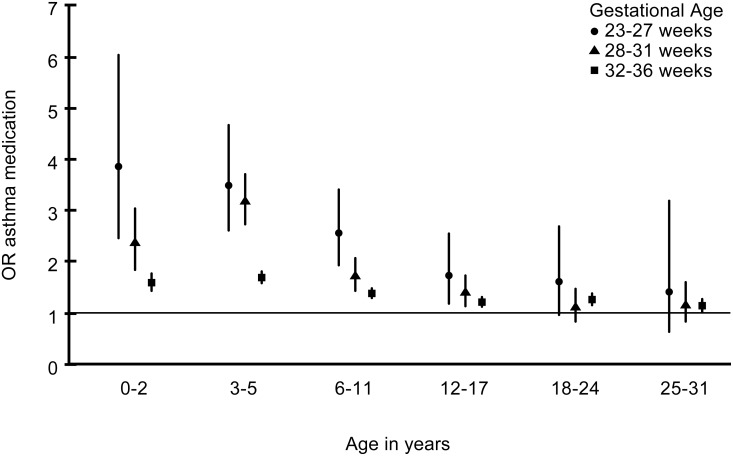
Unadjusted Odds Ratios (OR) within age groups for purchase of asthma medication in the three preterm groups. The term group is the reference.

Unadjusted ORs and 95% CIs (in parenthesis in the following) were above 1 for the preterm groups during childhood (age groups <18 years) with a dose-response relationship to GA (Fig. 3): They ranged from 1.59 (1.43–1.77) in 32–36 weeks’ GA to 3.86 (2.46–6.04) in 23–27 weeks’ GA for 0–2 years, and from 1.21 (1.12–1.31) in 32–36 weeks’ GA to 1.73 (1.18–2.55) in 23–27 weeks’ GA for 12–17 years. In adulthood ORs but not CIs remained above 1, except in the moderately preterm ie 32–36 weeks’ GA: 1.24 (1.19–1.29) at 18–24 years and 1.31 (1.25–1.38) in at 25–31 years.

Most covariates had stable associations with the outcome throughout all analyses (Table 3). Thus, no older siblings, cesarean section (both emergency and elective), maternal asthma medication, maternal atopy medication and acute neonatal respiratory disease all had ORs and CIs greater than 1 in all models. In the unadjusted analyses, emergency cesarean sections tended to present a comparable but slightly greater OR than elective sections. However, this relationship was reversed in all our adjusted analyses (the variable was not segregated in the oldest age group). Female sex ORs and CIs were lower than 1 in children, greater than 1 in adult age groups (18–31 years). Maternal smoking showed higher ORs and CIs in small children up to 5 years, but in age groups 6–17 years ORs were close or equal to 1. SGA mostly showed ORs greater than 1, but CIs did not show any consistency. BPD ORs and CIs were greater than 1 in our unadjusted analyses, except in the 18–24 years age group, and ranged from 1.43 (18–24 years) to 8.98 (25–31 years). In model 3, CIs did only persist above 1 for the 3–5 and 25–31 years groups. Multiple birth and maternal educational level did not show consistent associations with the outcome, but were kept in the models because of their known associations with gestational age and asthma-like symptoms in offspring.

**Fig 4:**
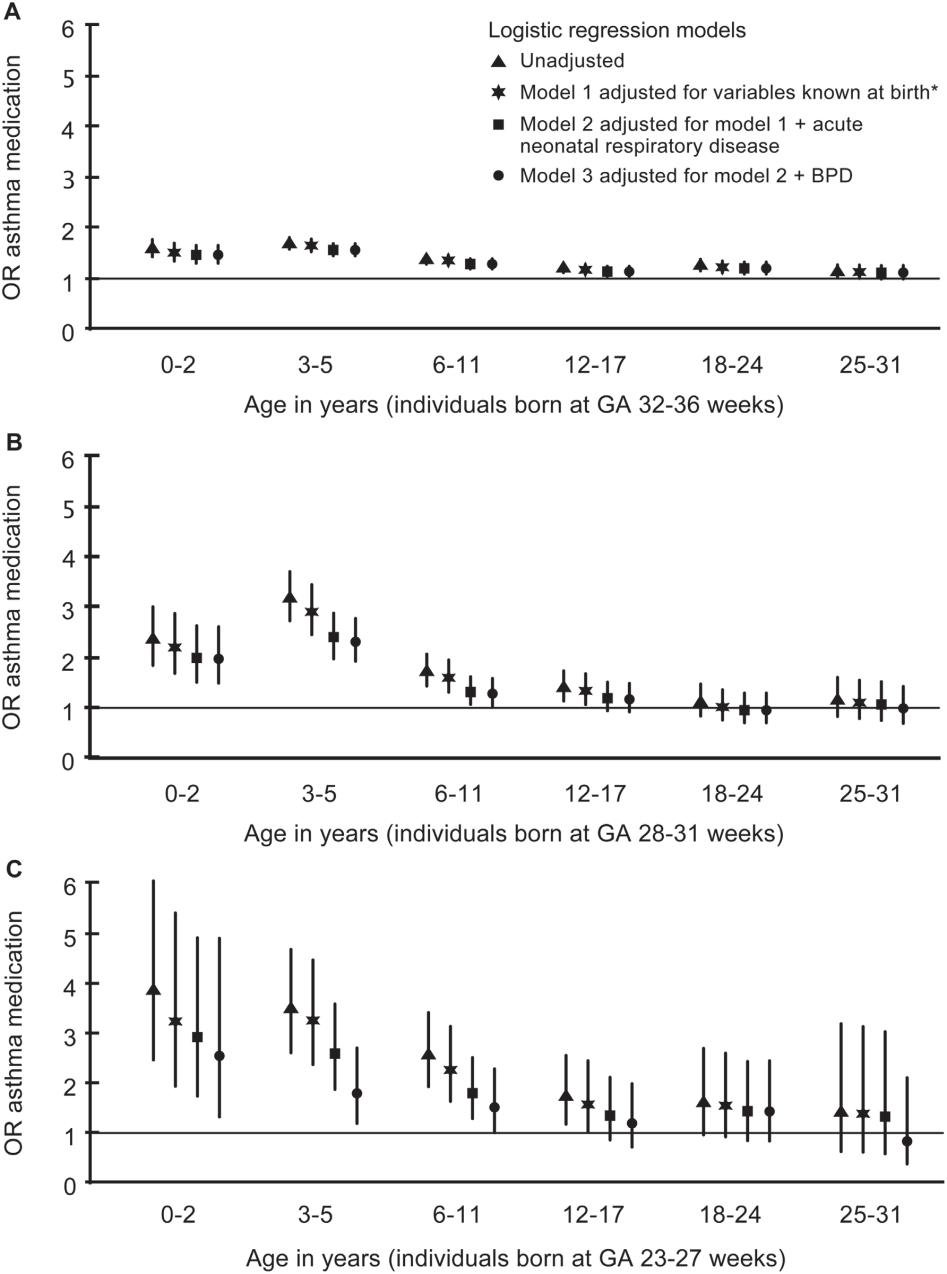
Odds Ratios (OR) within age groups for purchase of asthma medication in four regression models. ORs are presented in preterm groups: A (32–36 weeks), B (28–31 weeks) and C (23–27 weeks). The term group is the reference. (BPD: bronchopulmonary dysplasia). *variables known at birth: sex, SGA, older siblings, multiple birth, maternal educational level, cesarean section, maternal asthma medication, maternal atopy medication and maternal smoking (only available for age groups <18 years).

The results from the four different model setups are illustrated in Fig. 4. Adjusting for covariates present at birth had some effect in most GA groups and age groups. In the extremely preterm, adjusting for both potential mediators acute neonatal respiratory distress and BPD reduced ORs. In the very preterm group, the most important mediating factor seemed to be acute neonatal respiratory distress. In the moderately preterm, adjusting for covariates had little effect on ORs (Fig. 4).

## Discussion

### Interpretations

In this large long-term follow-up study, we found a strong association between gestational age at birth and purchase of prescription asthma medication in childhood. The association increased in a dose-response manner with decreasing gestational age, but weakened in higher age groups and was small or non-existent in adult groups.

In the moderately preterm group a weak association of GA with asthma medication persisted with ORs from 1.59 in infancy to 1.14 in adulthood. The fifty percent increased risk of purchasing prescription asthma medication in preschool children born moderately preterm is an important finding which also supports data from another recent study [20]. Our findings suggest that this risk is not solely related to neonatal respiratory distress. A recent register based Swedish study [9] found no increase in purchase of asthma medication in adults born moderately preterm. Although we found an increased risk of asthma medication purchase in these young adults, the 14% increase (from 2.1% to 2.4%) in prevalence is of uncertain clinical significance.

In the extremely preterm and very preterm groups, the associations of GA to asthma medication were strong in preschool-aged children. These associations weakened gradually in our school age and adolescent groups. In adulthood, ORs but not CIs remained above 1. Our findings corroborate the results of previous studies showing increased risk of airway disease in childhood in these individuals [8]. Our study design did not allow us to explore a possible treatment bias, i.e. that young children born extremely preterm are likely to receive asthma medication for non-specific airway symptoms, including virally induced wheeze, at a lower clinical threshold than children born at term. The precise nature of the respiratory symptoms in children born preterm is not clear although they are probably not due to classical atopic asthma. However, the increased purchase of prescription asthma medication still demonstrates a burden of persistent respiratory complaints. Several studies have documented a fixed airway flow limitation unaffected by asthma medication in individuals born preterm [3,5,21] and our results indicate a considerable clinical problem, as almost 20% of these preschool-aged children are affected. From school age and onwards, pulmonary lung function tests allow a more reliable diagnosis of asthmatic disease and treatment efficacy. This might partly explain the diminishing purchase of asthma medication after preschool age.

The choice of asthma medication varied in the GA-groups (S1 Table). Individuals born at term were more likely to receive combination inhalers, whereas individuals born preterm were more likely to receive short-acting β-2 agonists, inhaled glucocorticoids, and oral leukotriene receptor antagonists. Although not evidence based, this might reflect a prescription behavior where respiratory symptoms in individuals born at term would be considered more likely to be due to “true persistent asthma” than in individuals born preterm. However, our study was not designed to explain these variations.

We adjusted our multivariate analyses for neonatal respiratory morbidities, which we considered possible mediators, but these could not account for the full effect of prematurity. The association of prematurity per se with purchase of prescription asthma medication in our preschool- and school-aged children suggests that other factors associated with extreme and very preterm birth must also play an important role. Possible factors include disturbances in lung growth and alveolarization either due to the processes—including maternal risk factors–that lead to preterm birth and low birth weight or due to premature exposure to extrauterine conditions [22–25]. Furthermore, the ability of the preterm infant to recover from chronic lung disease may depend on the degree of postnatal alveolar “catch-up” growth achieved the first years of life [26] to puberty [27]. Finally, factors such as infections, aspiration, inadequate nutrition [28] and exposure to airborne pollutants including tobacco smoke in utero (and postnatally) [29] have all been associated with preterm birth and poor alveolar growth.

In the adult groups, the increased risk of asthma medication purchase in individuals born extremely and very preterm was not statistically significant. However, we believe it is important to bear in mind that extreme and very preterm birth is associated with increased airway responsiveness and some degree of pulmonary impairment in young adulthood [30]. It has been speculated that these individuals, whereas not requiring asthma medication in young adulthood, could still be at high risk of a relapse of obstructive lung disease later in life [31]. Further studies are needed to elaborate on the nature of this issue when more data from longer-term survivors are available [32].

Cesarean section was included as a possible exposure, confounder or mediator in our multivariate analyses. Previous studies have shown that emergency sections have a stronger association with purchase asthma medication than elective sections [33,34]. Our univariate analyses were partly in accordance with these findings, but our adjusted analyses showed a small reverse relationship. This might suggest that the variable is not just a marker of the physiological mechanisms involved in cesarean sections. In this case, it might rather be an indicator of maternal social factors or other environmental factors that have not been adjusted for in our multivariate analyses. The differences remained very small in our study.

### Methodological strengths and limitations

The interpretation of our findings is limited by the fact that the definition of BPD and the severity of the histopathological changes associated with BPD have changed over time [35,36]. A change from the ICD-8 to ICD-10 diagnose coding system in Danish healthcare was associated with an great increase in the prevalence of ‘BPD’ from 1994 onwards. The diagnostic criteria for coding are not very strictly defined in Danish clinical practice; they have changed during the data acquisition period and they have not been validated. A drastic change in treatment strategies towards a more restrictive approach to mechanical ventilation occurred in a large part of Denmark in 1987, which could explain some of the decrease in BPD prevalences from age group 25–31 (born 1980–1986) to 18–24 years (born 1987–1993). Advances in the treatment possibilities for preterm infants could also explain the slighter increase in acute neonatal respiratory disease as a result of improved survival rates. However, the cross-sectional aspect of our study did not allow us to adjust for these changes in treatment and their effect on survival rates. This makes it difficult, based on the results of our study, to predict the long-term risk of asthma or persistent asthma-like symptoms in extremely preterm infants being discharged from neonatal departments today. These limitations could explain why our results in the group of adults born extremely preterm differ from previous results [9].

Another register limitation is the lack of data on maternal smoking before 1991. However, the variable did not show significant associations with asthma medication from 6–17 years. Furthermore, associations between GA and purchase of asthma medication were not significant in the oldest age groups, with the exception of the moderately preterm. Finally, missing values don’t exceed 5% for this variable in each age group from 1991 onwards (S2 Table). Therefore, we estimated the effect of these missing data to be limited. Data on personal smoking history are not recorded in the Danish registers and were not available for the present study.

Some further limitations associated with our study design should be considered. First, the study is a cross-sectional evaluation of asthma medication purchase in different age groups. Thus, the decrease in prevalence in the older age groups may be a cohort effect: the younger age groups may have been more exposed to the real etiological factors that disturb lung growth and development compared to the older age groups. Prematurity per se, as well as both neonatal acute respiratory morbidity and BPD are possibly mere indicators of risk. Second, the study being register based, maternal prescription atopic medication was used as a proxy for atopic hereditary predisposition, which introduced a risk of misclassification of this variable. However, we found a prevalence of 17.9% in the maternal population comparable to prevalences of atopic eczema (17.7–22.4%) [37] and atopic rhinitis (23.1%) [38] in studies on similar Danish populations. We thus considered this variable a reasonable indicator of maternal atopic disease. Similarly, the prevalence of asthma-like symptoms, including asthma, was estimated through the purchase of prescription asthma medication and not based on lung function tests or physician diagnoses, since such data were not available through the Danish registers. Nevertheless, Moth et al. [39] found a high specificity (0.87, 95% CI: 0.85–0.88) for identifying Danish children with asthma through their purchase of asthma medication and our outcome prevalences in schoolchildren are similar to those of a recent study assessing physician-diagnosed asthma in Danish schoolchildren [40]. In order to study the more severe end of the spectrum of asthma-like symptoms, we specified asthma medication to include at least two purchases of other drugs for obstructive lung disease than β-2 agonists. Although these criteria lowered outcome prevalences, they hopefully contributed to increasing their reliability: behind the repeated purchase of prescription asthma medication lies a process of initial contact to a physician, a medical assessment of the problem leading to a prescription followed by at least two purchases. While repeated purchase does not prove continued use of the medication, it increases the likelihood of the medication actually being used. In Denmark, all access to primary care and prescriptions is free of charge. We did not have access to hospital pharmacy data, but the responsibility of asthma control lies with the general practitioners (GP). Therefore, asthma medication administered in hospitals is most likely limited to the treatment of severe asthma exacerbations requiring hospitalization. However, these individuals are probably followed-up—if not seen regularly—either by a GP or a specialist, from whom they are likely to receive prescriptions for regular treatment for their symptoms. These prescription medications can only be purchased at non-hospital pharmacies, and we are therefore confident that we have included these individuals in our analyses. Purchase of prescription medication is reimbursed from 0% to 50, 60, 75, 85 or 100% depending on age and annual medication expenses. Maternal educational level was the only variable assessing socioeconomic status. It is associated with preterm birth as well as with health seeking behavior and compliance. The variable, however, demonstrated only small and inconsistent associations with the outcome in our analyses. Therefore, it is unlikely that socioeconomic differences confounded our results significantly. Finally, the observational design of the study also introduces the possibility of other potentially significant confounders such as in vitro fertilization [41] and maternal antibiotic use [42], which were not included in the present study due to the establishment of the Danish National Prescription Registry in 1995. While our study does have limitations regarding the design, the definition of the outcome and covariates, the fact that it covers an entire population and the immunity to recall bias in routine registrations contribute to strengthen the generalizability of the results.

The strengths of this study are its large size and the high reliability of data from central databases individually linked by a unique CPR number, allowing investigation of the full spectrum of prematurity and several important confounders and mediators over a significant period of time. Furthermore, the register provenance of the analyzed data allows a minimization of selection and recall biases. Last, we believe that prescription of asthma medication in Denmark follows frequently updated national guidelines [43,44] with only relatively small differences between pediatricians and GPs, although poor adherence to guidelines cannot be excluded. Nonetheless, as previously indicated, purchase of bronchodilators as a single-drug therapy for mild non-specific airway symptoms was not included in the outcome. Likewise, repeated prescriptions and purchases are likely to increase the probability of our outcome variable representing a significant burden of airway disease. Clinically, the study thereby suggests that individuals born preterm outgrow their increased risk of asthma-like respiratory symptoms. This might imply either a “normalization” of their lung health [27] or less subjective attention to subclinical symptoms and/or mildly impaired lung function in early years of adulthood.

## Conclusions

We conclude that there was an association between gestational age and the purchase of prescription asthma medication in infancy and childhood, which appeared to weaken during adolescence and early adulthood. This association was only partly explained by neonatal respiratory morbidity diagnoses, indicating that other causal pathways exist. It remains to be investigated whether this weakening association occurs in longitudinal studies, and whether the strength of this association will increase again later in adulthood.

## Supporting Information

S1 Table Prescription asthma medication purchase and prevalences in % of ATC-groups in individuals meeting the outcome variable criteria by GA. GA: Gestational Age. ATC: Anatomical Therapeutic Chemical (classification system).
(DOCX)

S2 Table Missing values in each age group (crude numbers and %). (DOCX)
