# Supplementary material for: Prematurity and Prescription Asthma Medication from Childhood to Young Adulthood: A Danish National Cohort Study
Source: PLoS One. 2015 Feb 4;10(2):e0117253. doi: 10.1371/journal.pone.0117253 (PMC4317188; doi:10.1371/journal.pone.0117253)
Supplement: S1 Table — GA: Gestational Age. ATC: Anatomical Therapeutic Chemical (classification system). (DOCX) [file pone.0117253.s001.docx]

**Table S1. Prescription asthma medication purchase and prevalences in % of ATC-groups in individuals meeting the outcome variable criteria* by GA.**

|  | 23-27 weeks | 28-31 weeks | 32-36 weeks | 37-45 weeks | Total | p-values |
| --- | --- | --- | --- | --- | --- | --- |
|  |  |  |  |  |  |  |
| Study population | (n=2247) | (n=9626) | (n=84905) | (n=1693463) | (n=1790241) |  |
| At least one inhaled β-2 receptor agonist | 14.6 | 12.2 | 9.4 | 7.1 | 7.2 |  |
| Combination of at least one β-2 receptor agonist AND two other prescription asthma medications* | 8.0 | 5.9 | 4.5 | 3.1 | 3.2 |  |
|  |  |  |  |  |  |  |
| Individuals with outcome combination* | (n=180) | (n=571) | (n=3776) | (n=52880) | (n=57743) |  |
| Short-acting β-2 receptor agonists (R03AC02, R03AC03) | 92.8 | 91.9 | 89.9 | 86.9 | 87.2 | *<0.0001* |
| Long-acting β-2 receptor agonist (R03AC12, R03AC13, R03AC18) | 3.9 | 5.4 | 5.5 | 5.6 | 5.6 | 0.7964 |
| Combination inhaler (R03AK03**, R03AK04**, R03AK06, R03AK07) | 17.8 | 19.8 | 24.7 | 29.4 | 29.0 | *<0.0001* |
| Inhaled glucocorticoids (R03BA01, R03BA02, R03BA05, R03BA07) | 83.3 | 83.0 | 79.0 | 75.3 | 75.6 | *<0.0001* |
| Inhaled anticholinergics (R03BB01, R03BB04) | 0.6 | 0.5 | 0.3 | 0.3 | 0.3 | 0.4967 |
| Theophyllines (R03DA04, R03DA05) | 0.0 | 0.0 | 0.1 | 0.1 | 0.1 | 0.8182 |
| Oral leukotriene-receptor antagonists (R03DC03) | 27.8 | 25.7 | 22.6 | 20.4 | 20.7 | *<0.0001* |
| Systemic glucocorticoids (H02AB04, H02AB06, H02AB07) | 1.1 | 4.2 | 4.4 | 5.3 | 5.2 | 0.0029 |

GA: Gestational Age. ATC: Anatomical Therapeutic Chemical (classification system).

*Outcome was defined as either:

- a combination of at least one purchase of inhaled selective β-2 receptor agonist (R03AC) AND at least two purchases of one of the following other drugs for obstructive airway disease: inhaled glucocorticoids (R03BA), inhaled anticholinergics (R03BB), theophyllines (R03DA), oral leukotriene-receptor antagonists (R03DC), systemic glucocorticoids (H02AB), a combination inhaler (R03AK) OR

- at least two purchases of a combination inhaler (R03AK).

**Correspond to R03AL01 and R03AL02 respectively (β-2 agonists with anticholinergics) in current ATC-classification of 2014.
