# Supplementary material for: Prematurity and Prescription Asthma Medication from Childhood to Young Adulthood: A Danish National Cohort Study
Source: PLoS One. 2015 Feb 4;10(2):e0117253. doi: 10.1371/journal.pone.0117253 (PMC4317188; doi:10.1371/journal.pone.0117253)
Supplement: S2 Table — (DOCX) [file pone.0117253.s002.docx]

**Table S2. Missing values in each age group (crude numbers and %).**

|  | 0-2 years | | 3-5 years | | 6-11 years | | 12-17 years | | 18-24 years | | 25-31 years | | Total | |
| --- | --- | --- | --- | --- | --- | --- | --- | --- | --- | --- | --- | --- | --- | --- |
|  | (n=61492) | | (n=189215) | | (n=378430) | | (n=391192) | | (n=424955) | | (n=344957) | | (n=1790241) | |
|  | n | % | n | % | n | % | n | % | n | % | n | % | n | % |
| Older siblings | 732 | 1.2 | 2798 | 1.5 | 502 | 0.1 | 481 | 0.12 | 1 | 0.0 | - | - | 4514 | 0.3 |
| Multiple birth | - | - | - | - | 1 | 0.0 | - | - | - | - | 2 | 0.0 | 3 | 0.0 |
| Maternal educational level^1^ | 1229 | 2.0 | 2598 | 1.4 | 3147 | 0.8 | 4457 | 1.1 | 5805 | 1.4 | 4600 | 1.3 | 21836 | 1.2 |
| Cesarean section | - | - | 1 | 0.0 | 107 | 0.0 | 169 | 0.0 | 458 | 0.1 | 1946 | 0.6 | 2681 | 0.1 |
| Maternal asthma medication | - | - | - | - | - | - | 3 | 0.0 | 1 | 0.0 | 4 | 0.0 | 8 | 0.0 |
| Maternal atopic medication | - | - | - | - | - | - | 3 | 0.0 | 1 | 0.0 | 4 | 0.0 | 8 | 0.0 |
| Maternal smoking | 1389 | 2.3 | 4446 | 2.4 | 9565 | 2.5 | 19423 | 5.0 | ^2^ | ^2^ | ^2^ | ^2^ | 34823 | 3.4 |

^1^When maternal data were missing, paternal educational levels were used (in 2.1% of the cases in the variable). These cases were not considered missing.

^2^Not included in analyses for age groups 18-24 and 25-31. Variable was introduced in 1991.
